# Supplementary material for: Enhanced optical conductivity and many-body effects in strongly-driven photo-excited semi-metallic graphite
Source: Nat Commun. 2023 Nov 16;14:7407. doi: 10.1038/s41467-023-43191-5 (PMC10654445; doi:10.1038/s41467-023-43191-5)
Supplement: Supplementary file 1 — Supplementary Information [file 41467_2023_43191_MOESM1_ESM.pdf]

Supplementary Information –
Enhanced optical conductivity and many-body effects in strongly-driven photo-excited semi-metallic graphite

Supplementary Note 1: Experimental Setup

The experimental setup used is the same as in Ref. [1]. A 380-μJ, 1850-nm (0.7 eV), sub-2-cycle, CEP-stable pulse is sent into the beamline where it enters the Mach-Zehnder interferometer. Depending on the experiment, this pulse is split by a 95/5 (low fluence case) or 70/30 (high and medium fluence case) beam splitter and the low energy portion is directed as pump to the target. The high-energy part is focused into a high-pressure gas target, with backing pressures up to 12 bar in Helium. The condition for the present experiment was identical to the ones in Ref. [2] for producing an isolated 165-as pulse in the SXR water window with a spectrum from 250 to 500 eV. The emerging SXR pulse is sent through a 100 nm Sn filter to reject leftover infrared radiation before being focused onto target with a SXR ellipsoid (Carl Zeiss AG) to a target beam size of 15 ± 5 μm FWHM. After the target, a second 100 nm Sn filter is used to reject the pump light before the SXR radiation is dispersed with a home-built SXR spectrograph, which consists of a flat-field aberration-corrected and reflecting concave grating (2400 lines/mm; Hitachi High Technologies America, Inc.) and a cooled, back-illuminated CCD (PIXIS-XO; Princeton Instruments); the resolution of the spectrograph is 0.3 eV at the Carbon K-edge at 284 eV. The pump-probe delay is controlled via a closed-loop delay stage (Smaract GmbH) and the pump beam intersects the target at 2° with respect to the SXR beam. The pump beam focuses to 50 $\pm$ 1 μm FWHM, which is almost three times larger than the probe spot size, which ensures probing a homogeneously pumped region of the sample by the much smaller SXR beam. The basal plane of 95-nm free-standing graphite is oriented at 40 degrees with respect to the linearly p-polarized SXR pulse for the selective probing of bands with different orbital character. The pump is s-polarized to maximize the optical excitation from π to π* band.

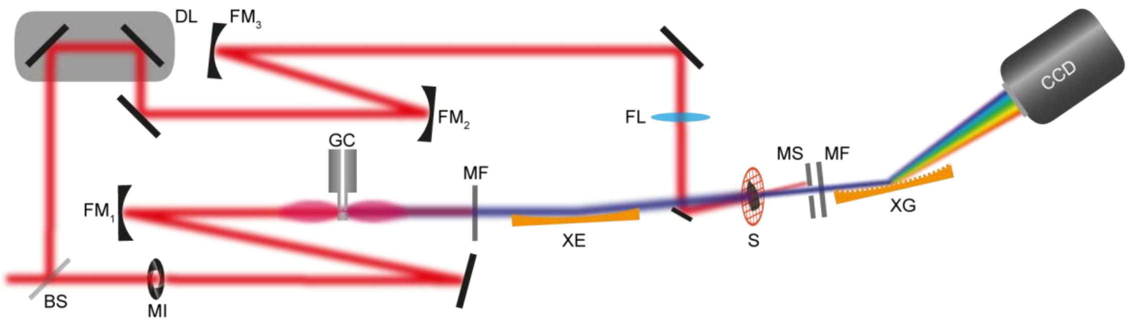

Supplementary Figure 1 Sketch of the pump-probe setup. At the input of the beamline the laser beam is separated by a beam-splitter (BS) into a pump and high-harmonic driver with a ratio depending on the desired pump fluence. The transmitted beam is focused (FM1) into a gas cell (GC) filled with Helium to produce isolated attosecond soft X-ray (SXR) pulses via high harmonic generation which constitute the probe of the experiment. The leftover infrared beam is then blocked by a metallic filter (MF) while the SXR pulses are transmitted and focused by an ellipsoidal mirror (XE) onto the thin graphite sample (S). The transmitted X-rays are dispersed by an X-ray grating (XG) and detected by an X-ray camera (CCD). The reflection of the beam-splitter constitutes the pump arm, synchronized with the probe with a delay line (DL). The beam is first expanded (FM2 and FM3), then focused with a calcium fluoride lens (FL, f = 500 mm) onto the sample, where the beam is overlapped in space and time with the probe. The remaining pump light is then blocked by a movable slit (MS) and a second metallic filter (MF) to reduce stray light on the camera.

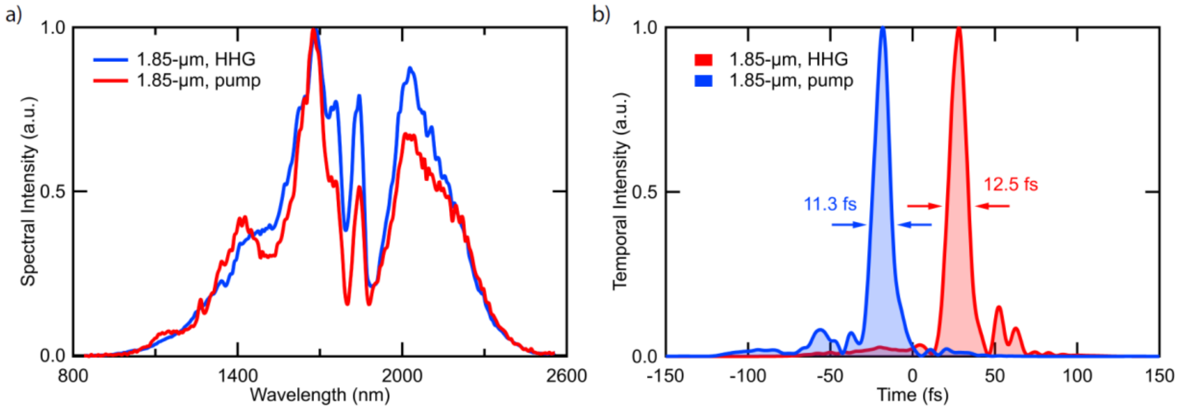

Supplementary Figure 2 Spectral and temporal characterization of the 1.85 µm pulse driving the HHG process and used as the pump pulse. **a,** Measured spectra for the 1.85 µm. Differences between the HHG driver and the 1.85 µm pump are due to the different optics used. **b,** Reconstructed temporal profiles from second-harmonic frequency resolved optical gating (SH-FROG) measurements.

**Pump parameter**	**1.85 µm (high)**	**1.85 µm (medium)**	**1.85 µm (low)**
**Fluence (vacuum) [mJ/cm^2]**	204 ± 13	57 ± 3.6	8.0 ± 0.5
**Intensity (vacuum) [TW/cm^2]**	18.0 ± 2.0	5.0 ± 0.3	0.55 ± 0.08
**E-field amplitude (vacuum) [V/Å]**	1.17	0.62	0.20
**Fluence (Graphite) [mJ/cm^2]**	81.4 ± 5	22.8 ± 1.4	3.2 ± 0.2
**Intensity (Graphite) [TW/cm^2]**	7.2 ± 0.1	2.0 ± 0.1	0.22 ± 0.03
**E-field amplitude (Graphite) [V/Å]**	0.37	0.19	0.07
**Ponderomotive energy [eV]**	10.8	3.0	0.4
**Keldysh parameter**	0.03	0.06	0.15
**Excited carrier density [10^{22} cm^{-3}]**	5.0	1.3	0.19
**Excited electrons/atom**	0.422	0.11	0.016

Supplementary Table 1 Pump pulse parameters

As described in the Method section we record 15 unpumped and pumped spectra in alternating order to reduce fluctuations originating from the HHG source. In Supplementary Fig. 3 we show that this procedure reduces intensity fluctuations below the observed absorption changes with no clear dependence on the pump-probe delay. To this end, for each delay we calculate the difference between the total unpumped and pumped counts in the signal region (280 eV – 300 eV) normalized to the mean of both.

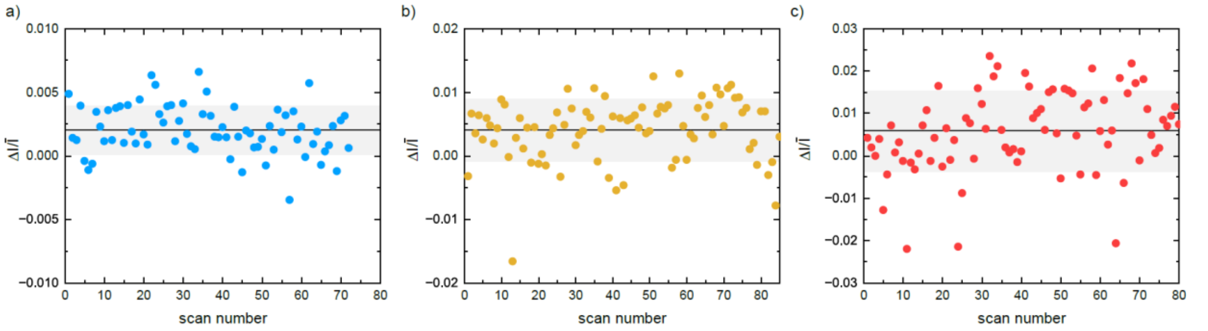

Supplementary Figure 3 Difference between the total unpumped and pumped counts, ΔI, in the signal region between 280 eV – 300 eV, normalized to the mean of both, $\bar{I}$, for the low fluence case in **a**, the medium fluence in **b**, and the high fluence case in **c**. The black solid line is the mean of all scans and the grey area the standard deviation of the mean.

To estimate the effect of source noise fluctuations on the observed absorption changes, we use the high fluence case as it provides the worst case with the largest noise. For each delay step we calculate a mean spectrum instead of summing all 15 frames. We then propagate the standard deviation of the mean spectra to obtain an error of the absorption changes for each recorded energy value and delay step as shown in Supplementary Fig. 4b. The error is greatest near the VHS around 286 eV, where absorption is strongest and thus the recorded transmitted spectrum is minimal. Supplementary Fig. 4c shows the lineouts from Supplementary Fig. 4a over the same energy range as used in Fig. 2d. The relaxation time obtained by fitting these lineouts matches, within the error, with the values obtained when calculating absorption changes by summing all recorded frames.

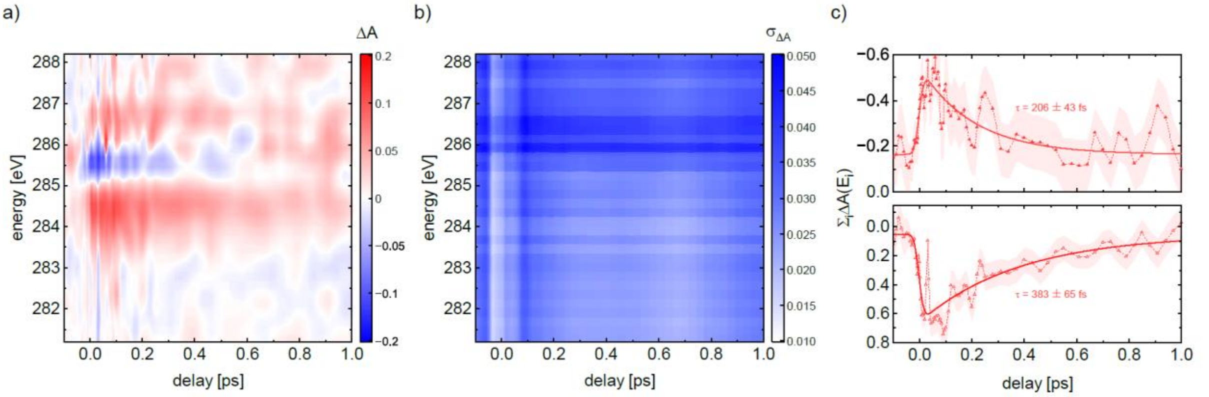

Supplementary Figure 4 Error estimation for ΔA. **a,** Absorption changes for the high fluence case as shown in Fig. 2c, however using the mean of 15 spectra instead of the sum. **b,** Error of the absorption changes by propagating the standard deviation of the mean of the 15 recorded spectra at each delay step. **c,** Lineouts from the absorption changes in **a** over the same energy range as used in Fig. 1d.

Supplementary Note 2: Optical excitation pathways

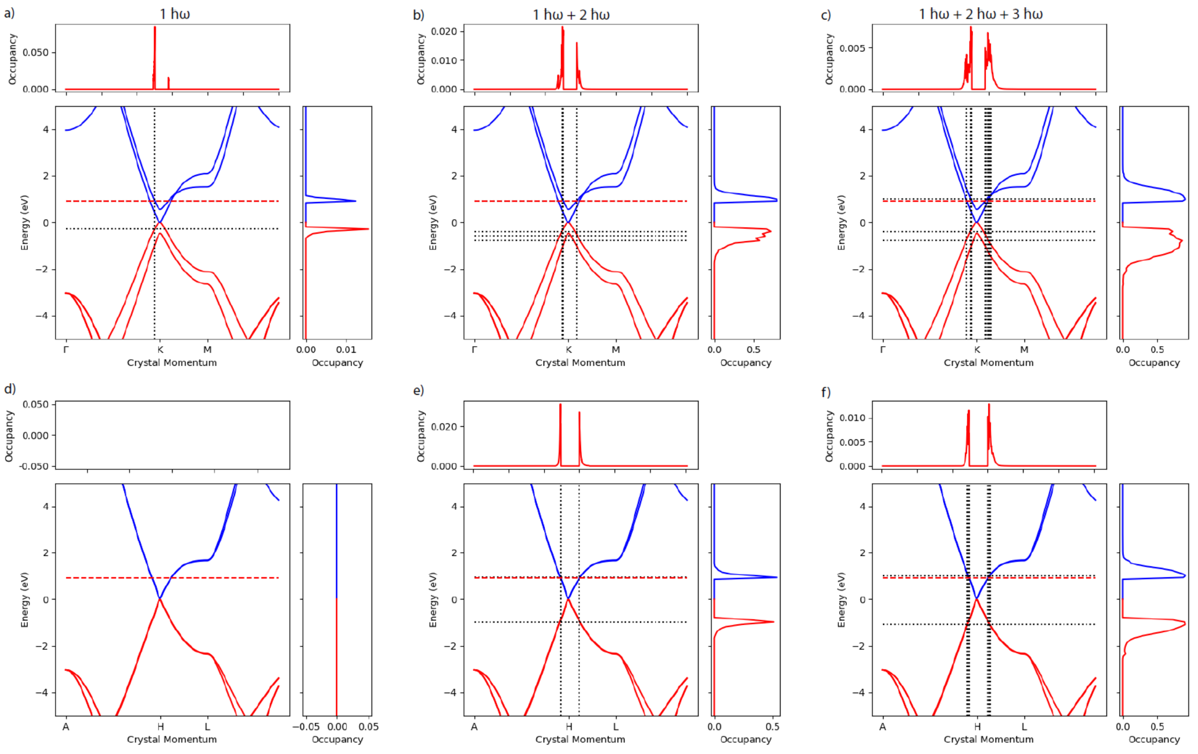

Supplementary Figure 5 Estimation of optically allowed excitation pathways from states with energies below, E_v, to states above, E_c, the Fermi energy (red dashed line) which lies 910meV above the K-point. Using the pump spectrum from Supplementary Fig. 2 we look for direct interband transitions which fulfil $E_c(k) - E_v(k) = n\hbar\omega$ for different crystal momentum k; in **a-c** along $\Gamma - K - M$ and in **d-f** along $A - H - L$. To obtain the 2- and 3-photon transition spectrum we scale the fundamental spectrum to its second and third harmonic energy. We assume equal transition probabilities for all states and only weight the transitions by the spectral amplitude. Vertical and horizontal dashed lines indicate peaks in the allowed transition spectrum.

Supplementary Note 3: Differential absorption trace

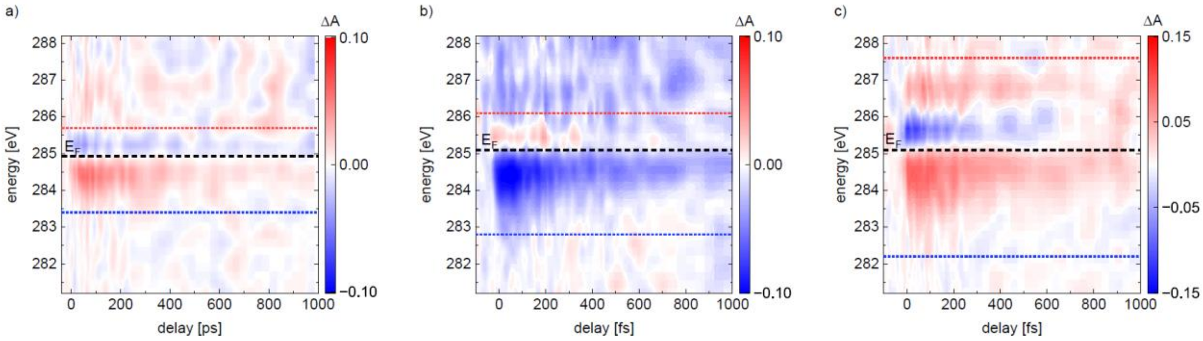

Supplementary Figure 6 Measured time-dependent differential absorption changes for the three fluences cases of 3.2 $\pm$ 0.2mJ/cm^2 (**a**), 22.8 $\pm$ 1.4mJ/cm^2 (**b**), and 81.4 $\pm$ 5 mJ/cm^2 (**c**). The black dashed line marks the position of the Fermi energy retrieved from the fit. The range between the Fermi energy and the red (blue) dashed line marks the energy region for lineouts for electrons (holes) in Figure 1d.

Supplementary Note 4: Fitting carrier occupation

The time- and energy-dependent absorption spectrum, $\mathrm{A}(E,t)$, is described by the product of calculated density of states, $\mathrm{DOS}(E,t)$, and a Fermi-Dirac distribution, $\mathrm{FD}(E,T(t))$. Changes to the DOS are accounted for by a shift, δ, and a stretching of the energy axis by a. Finally, the fitted spectrum is convoluted with a Voigt function, $\sigma(E,t)$, to account for the detector resolution through a Gaussian broadening of at least 250 meV, and the core-hole lifetime through a Lorentzian with a width of 150 meV [3],

$$A(E,t) = \sigma(E,t) * \left[1 - FD\big(E - E_F(t), T(t)\big)\right] DOS\big(a(t)\big(E - \delta(E,t)\big)\big) \qquad (1)$$

We separately apply this function to the unpumped and pumped spectra and minimize the difference between the fit and measured values at each recorded energy channel using a least-square method as implemented by the "lmfit" Python package [4]. The retrieved parameters then enable us to calculate the absorption spectrum with an interpolated energy axis and we obtain the thermalisation time by fitting the calculated time-dependant absorption spectrum by a single exponential decay shown on a log scale in Figure 2 of the main text and on a linear scale in Supplementary Fig. 11.

Fluence [mJ/cm^2]	σ [eV]	E_F [eV]	T [K]
3.2	0.355	284.9	320
22.8	0.357	285.1	232
81.4	0.343	285.3	200

Supplementary Table 2 Fit parameters for the unpumped spectra with a = 1, and $\delta = 0$

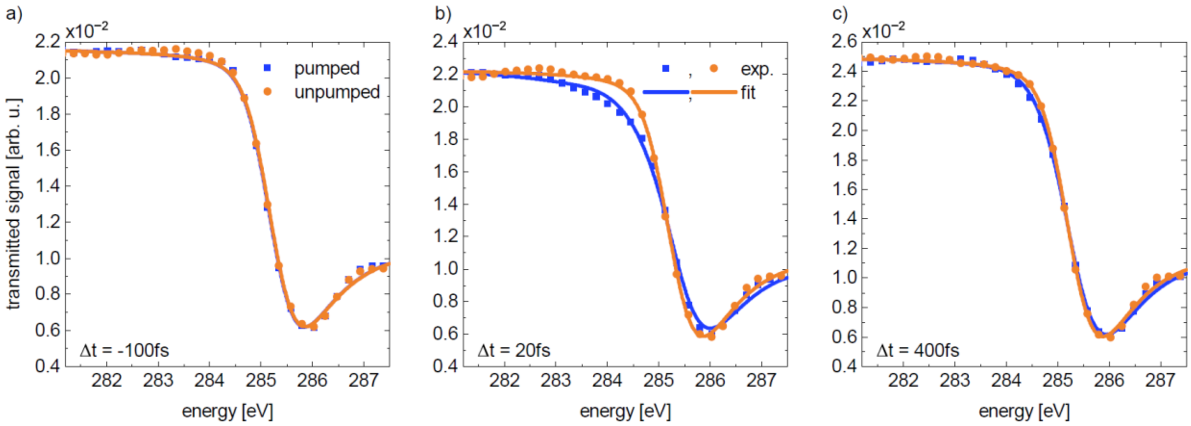

Supplementary Figure 7 Fits (solid lines) to the pumped (blue squares) and unpumped (orange circles) high fluence spectra for three selected delay times of -100 fs (**a**), 20 fs (**b**), and 400 fs (**c**). The transmitted spectra are normalised to the pre-edge region as described in the Method section.

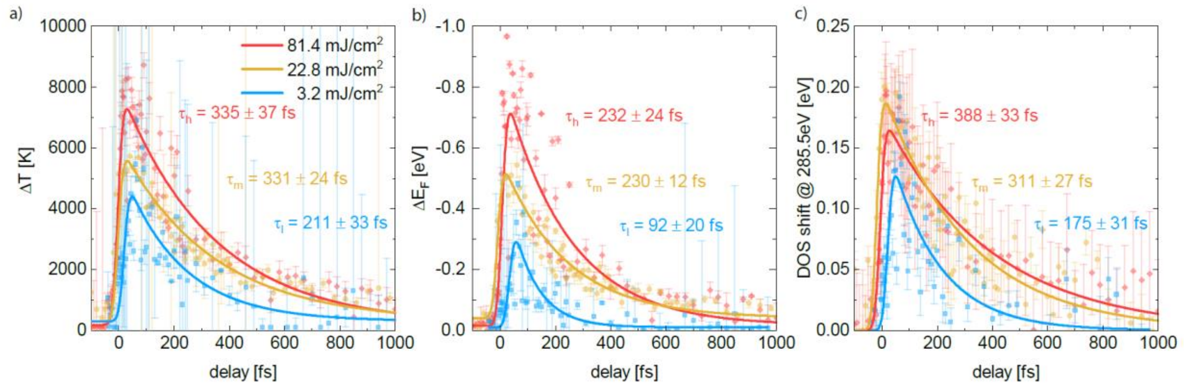

Supplementary Figure 8 Time-dependent parameters retrieved form the fit to the measured absorption spectra for the three fluences. **a,** Changes in the carrier temperature after optical excitation. **b,** shift of the Fermi-energy and **c,** shift of the DOS peak at 285.5eV.

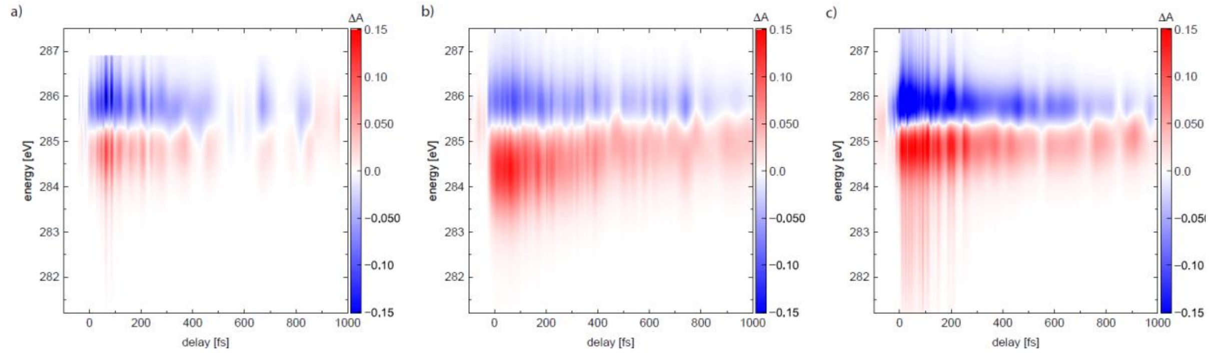

Supplementary Figure 9 Retrieved time-dependent change in absorbance, ΔA, for the three fluences of 3.2 ± 0.2mJ/cm^2 (**a**), 22.8 ± 1.4mJ/cm^2 (**b**), and 81.4 ± 5 mJ/cm^2 **(c)**, when only transient changes of the Fermi edge position and the carrier temperature are taken into account. To this end, each measured spectrum is first fitted with the model described by supplementary equation Eqn. 1, and then re-evaluated neglecting changes to the single particle spectrum, i.e. setting $a = 1$, $\delta = 0$.

In Supplementary Fig. 8 we show the retrieved carrier temperature, Fermi edge position, and the shift of the DOS for the three fluences of 3.2 ± 0.2 mJ/cm^2, 22.8 ± 1.4 mJ/cm^2 and 81.4 ± 5 mJ/cm^2. The relaxation times are obtained from a fit with an exponential convoluted by a Gaussian to account for the excitation with the 11.3-fs long NIR pump pulse. Similar to previous works on time-resolved absorption spectroscopy from core-states, we included a time-dependency of the Gaussian broadening in the fits, however we find best convergence when both pumped and unpumped spectra have the same broadening [5,6].

For low photo-doping with 3.2 ± 0.2 mJ/cm^2 carriers reach a peak temperature of 4000 K and thermalize within 211 ± 33 fs. With an increase to 22.8 ± 1.4 mJ/cm^2 carriers become hotter (5500 K) and require longer to cool (331 ± 24 fs). While an increase in photo-doping with 81.4 ± 5 mJ/cm^2 further raises carrier temperatures to 8000 K relaxation times stay similar (335 ± 37 fs). We want to emphasize that this temperature is an average throughout the sample and can reach values above 10000 K at the front surface.

In graphene the slow relaxation of photo-doped carriers is a consequence of the low density of states near the K-point, the large optical phonon energies, and the slow cooling of optical phonons through phonon-phonon scattering [1,7–9]. At high fluences in the mJ/cm^2 regime ultrafast carrier-carrier

scattering establishes a hot carrier distribution with a temperature, which, in our case, is close to the photon energy. Band filling due to the low density of states near the K(H)-point and the large optical phonon energies limit the relaxation of low energetic carriers [9,10]. For carriers with energies larger than the optical phonon energy, relaxation through the strong coupling of electrons to optical phonons becomes possible, rapidly increasing phonon temperatures. Consequently, the slow cooling of the hot optical phonon distribution limits carrier relaxation [1,11]. For the high fluence case, another mechanism has to be considered as carriers are excited into states near the VHS where the density of states is large and bands become flat. The flat-band states require carriers to transfer a large momentum in order to significantly lose energy. Thus, carrier thermalisation through carrier-carrier scattering is supressed and scattering with strongly coupled optical phonons becomes the main relaxation channel.

Similar to the carrier temperature the relaxation of the Fermi edge does not change when increasing the fluence to 81.4 ± 5 mJ/cm^2, however recovers about 100fs faster suggesting a different relaxation mechanism, see Supplementary Fig. 8b. The edge shift of about 0.8 eV towards the K-point within 30 fs after excitation by the pump pulse indicates a significant imbalance between electrons and holes that highlights the efficient hot carrier multiplication in highly n-doped graphite occurring on ultrashort time scales [1,8,12]. In the first 100 fs after optical excitation holes are efficiently generated through impact ionization, shifting the Fermi edge towards the K-point. For later time scales, however, Auger recombination becomes dominant balancing the carrier population while efficiently transferring energy to other carriers. Thus, the generation of hot carriers through Auger recombination slows down carrier cooling with respect to the relaxation of the Fermi edge.

Following the discussion on the carrier occupation described through the Fermi-Dirac distribution we now turn to the time-dependent scaling of the single-particle electronic structure and its response to the optically excitation with the NIR pump pulse. In Supplementary Fig. 8c we show the renormalisation of the DOS by tracking the VHS after optical excitation. It is this shift that to leads to the additional positive absorption change above 286 eV in Fig. 1c of the main text. For early times the DOS rapidly shifts to higher energies by nearly 200 meV which is in contradiction to the previously reported photo-induced band-gap renormalization in graphite [13–15] and other layered semiconductors [16,17]. Instead, the large pump photon induced carrier density before the arrival of the SXR probe effectively screens the 1s core-hole state that causes a blue-shift of the pi* peak in the SXR absorption spectrum [18–20]. The relaxation dynamics of this peak shift is therefore similar to that of the carrier temperature as only through carrier cooling and recombination core-hole screening is reduced and the absorption spectrum approaches that of unexcited graphite.

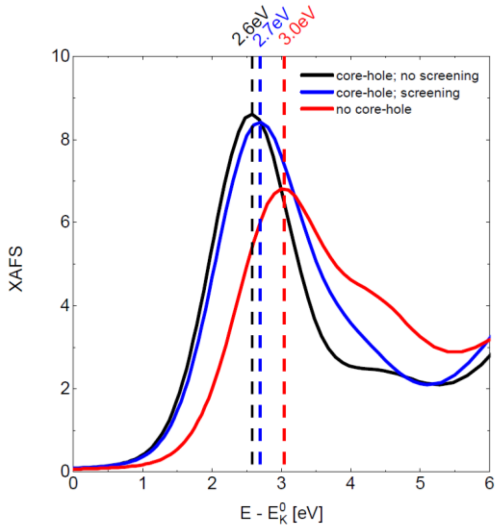

Supplementary Figure 10 Simulated absorption spectrum of graphite with an unscreened core-hole (black), a screened core-hole (blue), and without a core-hole (red). The dashed lines mark the peak positions of π^* peak.

We use the FDMNES packages to simulate the SXR absorption spectrum of graphite and study the influence of core-hole screening on the position of the π^* peak in the absorption spectrum [21]. First, the absorption spectrum with an excited core-hole is red-shifted by 440 meV compared to the peak position when no core-hole is excited. Now, introducing a screening of the core-hole, the absorption peak blue-shifts and is only 330 meV lower in energy than for the case when no core-hole is present. This, blue-shift of the absorption spectrum introduced by the screening of the core-hole agrees with the observed shift in experiments where optically excited charge carriers screen the core-hole and as such the shift is linked to carrier dynamics rather than core-hole dynamics.

Supplementary Note 5: Carrier thermalisation

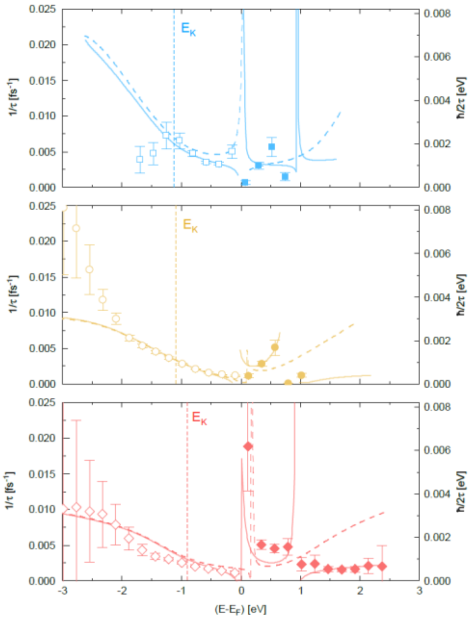

Supplementary Figure 11 Comparison of retrieved thermalisation rate $1/\tau$ for electrons (filled symbols) and holes (open symbols) from experiment and the fit to the absorption spectra for a static DOS (dashed lines) and with renormalization of the DOS (solid lines).

In Fig. 2 of the main text we present the carrier thermalisation rate on a log-log scale to highlight the dimensionality of the carrier system. By plotting the carrier thermalisation on a linear scale in Supplementary Fig. 11, we highlight the similarity of the thermalisation rate to the self-energy of n-doped graphene; in particular, when no renormalization of the DOS is present. Therefore, after fitting the absorption spectra for each time delay with the above model, we set $a(t) = 1, \delta(t) = 0$, and retrieve the thermalisation rate for a static DOS from exponential fits to $A(E,t)$. The corresponding transient absorption changes are shown in Supplementary Fig. 9.

Supplementary Note 6: Scaling behaviour of the DOS with energy

The scaling of the density of states with energy can be derived from the volume of a single electronic state in a n-dimensional system and the dispersion relation of the electron. In the table below we show the scaling of the DOS for a linear dispersion ($E = \mathrm{v_F}\hbar k$; p = 1), as it is the case near the K- and H-point, and for a quasi-free electron with a quadratic dispersion ($E = \frac{\hbar k^2}{2m^*}; \mathrm{p} = 2$). For a carrier dispersion with a vanishing slope (p = 0), a classical derivation of the DOS would yield a Dirac-Delta function in n-dimensions. However, the exact scaling of the DOS depends on the type of flat-band as defined by the curvature of the carrier dispersion. In graphite, the carrier dispersion vanishes at the extrema near the K-point and near the M-point, where no simple scaling laws for the density of states are possible. We refer to the literature for a detailed discussion on the scaling of the density of states near extrema in the carrier dispersion in graphite [15,22] and for flat bands in general [23,24].

	n=1	*n=2*	*n=3*
p = 1	$\frac{2}{\pi\hbar v_F}$	$\frac{1}{v_F^2\hbar^2}(E - E_0)$	$\frac{1}{\pi^2 v_F^3\hbar^3}(E - E_0)^2$
p = 2	$\frac{1}{\pi\sqrt{\frac{1}{2m^*}}\hbar}(E - E_0)^{-\frac{1}{2}}$	$\frac{1}{2\pi\left(\frac{1}{2m^*}\right)\hbar^2}$	$\frac{1}{2\pi^2\left(\frac{1}{2m^*}\right)^{\frac{2}{3}}\hbar^3}(E - E_0)^{\frac{1}{2}}$

Supplementary Table 3 Scaling of the density of states for different spatial dimensions n and scaling of the carrier dispersion p.

Supplementary References

[1] T. P. H. Sidiropoulos *et al.*, Phys. Rev. X **11**, 041060 (2021).
[2] S. L. Cousin, Phys. Rev. X **7**, 041030 (2017).
[3] K. C. Prince *et al.*, Phys. Rev. B **62**, 6866 (2000).
[4] M. Newville *et al.*, (2014).
[5] M. Zürch *et al.*, Nat. Commun. **8**, 15734 (2017).
[6] A. R. Attar *et al.*, ACS Nano **14**, 15829 (2020).
[7] D. Yadav, M. Trushin, and F. Pauly, Phys. Rev. B **99**, 155410 (2019).
[8] D. Brida *et al.*, Nat. Commun. **4**, 1987 (2013).
[9] S. Winnerl *et al.*, J. Phys.: Condens. Matter **25**, 054202 (2013).
[10] T. Winzer and E. Malic, J. Phys. Condens. Matter **25**, 054201 (2013).
[11] A. Stange *et al.*, Phys. Rev. B **92**, 184303 (2015).
[12] E. Malic *et al.*, Phys. Status Solidi B **253**, 2303 (2016).
[13] M. Breusing, C. Ropers, and T. Elsaesser, Phys. Rev. Lett. **102**, (2009).
[14] S. Pagliara *et al.*, J. Am. Chem. Soc. **133**, 6318 (2011).
[15] P. Rosenzweig *et al.*, Phys. Rev. Lett. **125**, 176403 (2020).
[16] A. Faridi, D. Culcer, and R. Asgari, Phys. Rev. B **104**, 085432 (2021).
[17] M. Zapf, T. Sidiropoulos, and R. Röder, Adv. Opt. Mater. **7**, 1900504 (2019).
[18] M. Dendzik *et al.*, Phys. Rev. Lett. **125**, 096401 (2020).
[19] R. Buczko *et al.*, Phys. Rev. Lett. **85**, 2168 (2000).
[20] A. Zunger, Phys. Rev. Lett. **50**, 1215 (1983).
[21] S. A. Guda *et al.*, J. Chem. Theory Comput. **11**, 4512 (2015).
[22] C. Bena and S. A. Kivelson, Phys. Rev. B **72**, 125432 (2005).
[23] Y. Kulynych and D. O. Oriekhov, Phys. Rev. B **106**, 045115 (2022).
[24] N. F. Q. Yuan, H. Isobe, and L. Fu, Nat. Commun. **10**, 5769 (2019).
